# Otology versus Otosociology

**DOI:** 10.5402/2012/145317

**Published:** 2012-10-30

**Authors:** Miguel A. Lopez-Gonzalez, Georgina Cherta, Jose A. Nieto, Francisco Esteban

**Affiliations:** ^1^UGC Otorhinolaryngology, Virgen of Rocio University Hospital, C/Manuel Siurot, s/n 41.013 Sevilla, Spain; ^2^Faculty of Political Sciences and Sociology, National University of Distance Education, 29.006 Malaga, Spain; ^3^Faculty of Political Sciences and Sociology, University of Madrid, 28.080 Madrid, Spain

## Abstract

Otology concerns the biological study of ear alterations and diseases, solely. So, the diagnosis of audiovestibular diseases tends to be idiopathic or is based on theoretical concepts such as idiopathic sudden deafness, Ménière disease, benign paroxysmal positional vertigo, tinnitus, hyperacusis, or idiopathic facial paralysis. The treatment for these pathologies is symptomatic. Otosociology takes the aetiology and pathogenesis of the ear and situates them within the social and cultural environment of the patient. Then, audiovestibular disease is based on evidence, and the treatment options seek to solve the causes and consequences produced. Otosociology should be considered as a new discipline. Otosociology came into being since otology does not provide definitive solutions for the audiovestibular alterations produced from the point of view of the ear, whereas otosociology finds these solutions within the social/cultural environment of the patient. Where otology emphasises the diseases of the ear, otosociology deals with social manifestations. Where otology deals with idiopathic diseases, otosociology deals with causes and pathogeny produced by interactions in the social and cultural surroundings of the patient. Where otology offers symptomatic treatment, otosociology offers treatment of causes and consequences. Otosociology can fill significant voids in audiovestibular processes from the perspective of the patient's social environment.

## 1. Introduction

Quite often, we come across diseases in various medical specialties that are catalogued in biomedicine as idiopathic, that is to say, diseases with no apparent or known cause. Normally, these idiopathic diseases are treated with symptomatic remedies, whose objective is to improve the symptoms that have negative impacts on the normal lifestyle of patients, but without dealing with or solving the causes that provoke them. Biomedicine is always searching for the causes for diseases inside the organism, without finding them, when the causes and pathogenesis tend to be outside of the organism, in the environment. Often, treatment consists of social modifications that attempt to resolve these pathological processes. Environmental factors influence all diseases, but in idiopathic processes are crucial.

Given the limitations of biomedicine to give clear explanations, and consequently a cure or remedy for certain diseases, a new paradigm is needed that can explain the causes of these pathologies that are considered idiopathic. To this end, it is essential that we integrate different elements through the formation of collaborative groups or “health teams,” defined by the World Health Organisation in 1973 as “a nonhierarchical group of people with different professional backgrounds but a common objective, which is to provide the most comprehensive care possible to patients and their families, in any situation.” Currently, collaborative work teams can be found in several different medical specialties such as oncology, geriatrics, and forensic medicine, whose health teams are primarily composed of health care professionals. One striking exception is radiology and traumatology, in which other specialties are starting to be incorporated such as biomedicine, physics, and engineering.

Within otorhinolaryngology, a medical/surgical specialty that is concerned with the prevention, diagnosis, treatment, and rehabilitation of diseases of the ear and upper respiratory/digestive tracts (mouth, nose, pharynx, and larynx), and the functions derived from these structures (hearing, respiration, olfaction, taste, swallowing, and phonation: voice and speech) as well as the cervical and facial structures connected or related to these pathologies and sociology, a science dedicated to the empirical and theoretical analysis of social processes and structures. More specifically, it is the close collaboration between otology, which involves the biological study of diseases and abnormalities of the ear, and health sociology that “directly collaborates with doctors and other health professionals” [1], in addition to the syncretic integration of other disciplines such as anthropology and social/clinical psychology.

 In this manner, the joint labour of otology and sociology gives way to otosociology, a discipline dedicated to “the study, intervention, and prevention of organic and functional pathologies of the auditory system with special emphasis on the influence of social factors.”

 In the following sections, we describe how otosociology is capable of explaining both the social consequences and causes of certain diseases identified by otology as idiopathic. In the following section (diseases, ischaemia, and alterations), we describe the process of passing from identification of audiovestibular diseases recognised by otology to discuss these abnormalities as symptoms from the viewpoint of otosociology. In the third section (otosociology), we explain both the training and work focus of otosociologists and the methodologies employed by this new perspective, justifying its use in daily otorhinolaryngological practice. Finally, we finalise the conclusions that have been made.

## 2. Diseases, Isquemia, and Alterations

Until now, audiovestibular pathologies have been treated by otological medicine, which identifies them exclusively as biological diseases, attempting to situate the aetiopathogenesis in the audiovestibular organ itself, and as a result, the causes and consequences remain hidden, making any treatment strategy strictly palliative in nature. In contrast, otosociology views and treats these pathologies as symptoms of a social problem that affects the biological part of the subject. Otosociology, by identifying social problems that cause these symptoms and alterations in the patient's environment, can apply effective medical treatment and directly address the social consequences (Table 1).

### 2.1. Audiovestibular Diseases in Otology

The most important otological pathologies are sudden deafness, Ménière disease, benign paroxysmal positional vertigo, tinnitus, and hyperacusis, commonly grouped within the category of audiovestibular diseases. These diseases cause patients to seek otological care and are immediately ascribed to the ear and are produced by the ear and are treated as exclusively otic pathologies. Many examples of the aetiology, incidence, diagnosis, treatment, and prognosis of these audiovestibular diseases are available in the medical literature and are discussed here.

#### 2.1.1. Sudden Deafness

Sudden deafness was first described as a disease by De Kleyn in 1944 [2]. Its otological definition is sensorineural or perceptive hearing loss, usually in one single ear, of sudden onset, with a loss of over 30 dB, at least three consecutive frequencies, with no previous otological background. 

 Otology attempts to discern the causes of sudden deafness in the ear, and several aetiologies have been proposed such as rupture of the cochlear membrane, microangiopathic processes in the ear, viral ear infection, autoimmune diseases of the inner ear, Ménière disease, vestibular schwannoma, or meningioma [3], although none of these theories sufficiently clears up the issue, nor can be applied in all cases.

The incidence of sudden deafness has increased over time and is estimated to reach 160 cases per year per 100 000 inhabitants [4]. In Japan, where sudden deafness is registered [5], probable causes of the increase in sudden deafness include increased general awareness of this disease in the Japanese population and the presence of diseases correlated with lifestyle, such as hypertension, diabetes, hyperlipidemia, and heart disease, associated with vascular pathologies, with the conclusion that vascular pathologies derived from hypertension and diabetes can lead to alterations in cochlear microcirculation, which leads to sudden deafness from cellular stress.

This diagnosis is reached through clinical symptoms, audiometry, and a magnetic resonance of the internal auditory canal through which the auditory nerve passes. The diagnosis is idiopathic sudden deafness.

Medical treatment, which is an idiopathic process, is based on corticosteroids, vasodilators, and antioxidants.

The patients with the worst potential prognosis for recovery are those with old age, severe initial hearing loss, vestibular symptoms, late treatment and time to recovery (the longer it takes to recover, the greater the chance that the patient never will), and the presence of tinnitus (Table 2). 

#### 2.1.2. Ménière Disease

In otology, Ménière disease is defined as an internal ear disorder that affects both balance and hearing, characterised by an abnormal sensation of movement or rotatory vertigo, loss of hearing in one or both ears, tinnitus, sensation of aural fullness, and hyperacusis and occurs in recurring crises. Ménière in 1861 [6] described in his “*Mémoire sur des léssions de l'oreille interne donnat lieu à des symptómes de congestion cérébrale apopectiforme”* the findings from an autopsy of a woman, in which he observed damaged semicircular canals full of a red, plastic material, resembling a sort of bloody exudation that was only marginally present in the vestibule and nonexistent in the cochlea. Seven years after the death of Ménière, his student Politzer (1867 cited by Rizzi in 2000) [7] published these symptoms as Ménière disease in the Archives für Ohrenheilkunde. Twelve years after the death of Ménière, Charcot (1874 cited by Baesly and Jones, 1996) [8] popularised the name of Ménière disease for the symptoms of vertigo, deafness, and tinnitus.

Ménière disease affects the inner ear with an unknown aetiology, characterised by a dilation of the membranous labyrinth due to increased endolymph (endolymphatic hydrops) of an unknown cause. The incidence of this disease ranges between 17/100 000 in Japan [9] and 205/100 000 in Italy [10].

Ménière disease is clinically diagnosed when the patient develops recurrent crises of rotatory vertigo, low-frequency fluctuating sensorineural hearing loss, hyperacusis, and a sensation of blockage in the ear or aural fullness. Several major efforts have attempted to establish unified diagnostic criteria at the international level. The Committee on Hearing and Equilibrium of the American Academy of Otolaryngology-Head and Neck Surgery (1995) [11] put together a guideline based on clinical histories with four stages: (1) possible Ménière disease (episodes of vertigo with no hearing loss, fluctuating or fixed sensorineural hearing loss, with disequilibrium but no definitive episodes, excluding other possible causes). (2) probable Ménière disease (one episode of vertigo; audiometrically documented hearing loss on at least one occasion; tinnitus or otic pressure), (3) definite Ménière disease (two or more episodes of vertigo lasting at least 20 minutes; audiometrically documented hearing loss on at least one occasion; tinnitus or aural fullness of the affected ear), and (4) certain Ménière disease (established disease with histological confirmation). Since biopsy is not possible without destroying the inner ear, confirmation is only possible through autopsy; that is to say, no living patient has been diagnosed with certain Ménière disease.

In Ménière disease, the worst symptom for the patient is vertigo, requiring medical treatment with corticosteroids, benzodiazepines, dimenhydrinate, thiethylperazine, or sulpiride. If these medical treatments fail, drugs such as corticosteroids and gentamycin can be administered directly into the inner ear. Another therapeutic option is pressotherapy which places pressure on the middle ear that in turn affects the inner ear and can improve the vertigo by affecting the pressure exerted on the liquids in the inner ear [12]. The final alternative for the treatment of vertigo can involve a neurectomy of the vestibular nerve, a labyrinthectomy, or drainage of the endolymphatic sack. The prognosis varies between mild discomfort and incapacity (Table 3).

#### 2.1.3. Benign Paroxysmal Positional Vertigo

Benign paroxysmal positional vertigo is defined as a situation in which brief episodes of vertigo are produced by movements of the head. These episodes of vertigo spontaneously disappear and are of frequent and unpredictable recurrence. This process was described for the first time in 1921 by Bárány [13]. The incidence of this condition is estimated at 46–81 cases per 100 000 inhabitants and increases by 38% for every decade of life [14]. The idiopathic variety is twice as common in women as in men and occurs between the ages of 50 and 70 years [15, 16]. When the aetiology is trauma or vestibular neuritis, no such differences appear between the sexes [17]. In elderly individuals it may go unnoticed in daily life and only is recognised when undergoing diagnostic tests [18].

Schuknecht (1962) [19] and Schuknecht (1969) [20] proposed the theory of cupulolithiasis to explain how this vertigo is produced within the inner ear. According to this theory, this vertigo is caused by microscopic stones composed of calcium carbonate and proteins, otoliths, which move within the utricle of the otic vestibular system, that is to say, the interior of the equilibrium centre. For their part, Hall and colleagues (1979) [21] proposed the theory of canalithiasis, stating that these minute particles circulate improperly through the canals of the inner ear labyrinth, altering balance and producing vertigo. Dix and Hallpike (1952) [22–24], who had thoroughly researched vertigo of the ear, developed a diagnostic test for this process, the Dix-Hallpike test.

Thus, once the otic mechanisms of this vertigo and how to diagnose it were established, Semont and colleagues [25] in 1988 established the treatment of a repositioning manoeuvre to place the calcium deposits in their original place in order to halt the vertigo, known as the Semont manoeuvre. In a similar manner, in 1992, Epley [26] described another repositioning manoeuvre for the posterior canal, known as the Epley manoeuvre. Recently, Hilton and Pinder (2002, 2004) [15, 16] performed a review in the prestigious Cochrane organisation in which they demonstrate that the Epley manoeuvre is effective at repositioning the calcium deposits in the inner ear. The prognosis, from an otological point of view and as its definition indicates, is benign, recurrent but benign.

As has been shown, the diagnosis of benign paroxysmal positional vertigo has a perfectly defined set of signs and symptoms, which are always produced, diagnosed, and treated within the inner ear (Table 4).

### 2.2. Ischaemia as an Explanation for Audiovestibular Processes

Ischaemia is a deficit of blood flow, whether transient or definitive, in an organ or part of it. The concept of ischaemia allows us to make a significant conceptual advancement since the medical viewpoint of the condition starts to look outside of the ear when focusing on its vascularisation, although other factors may be interacting.

In recent years, several scientific advancements have been made in this field. With regard to sudden deafness, the ischaemic processes of the inner ear have arisen as a mechanism of pathogenesis [27]. In Ménière disease, Pirodda et al. [28] proposed a model based on haemodynamic disequilibrium that produces transient ischaemia and could have an effect on pH and the proton pump of the inner ear. In this manner, under conditions of ischaemia and metabolic acidity, the activity of the proton pump would create an overload of anions in the endolymph with the result of increased osmolality. This, in turn, leads to the formation of hydrops, an increase in pressure in the endolymph fluid, which causes the fluctuating deafness, vertigo, and tinnitus that are characteristics of Ménière disease. It has also been established that endolymph hydrops can be produced without causing vertigo [29–33]. Similarly, vertebrobasilar ischaemia is starting to be considered as the pathogenesis of benign paroxysmal positional vertigo [34]. To conclude, it is interesting to point out that currently, several authors consider ischaemia of the nervous system as the pathogenic mechanism of tinnitus [35–41], a symptom produced in the majority of cases of sudden deafness and Ménière disease.

This simple change in perspective allows us to extend our focus outside of the auditory organ, a structure specific to the field of otology, in order to offer explanations of these otic pathological processes, favouring the examination of ischaemia as a pathogenic factor and passing from an otological viewpoint to that of otosociology.

**Figure 1:**
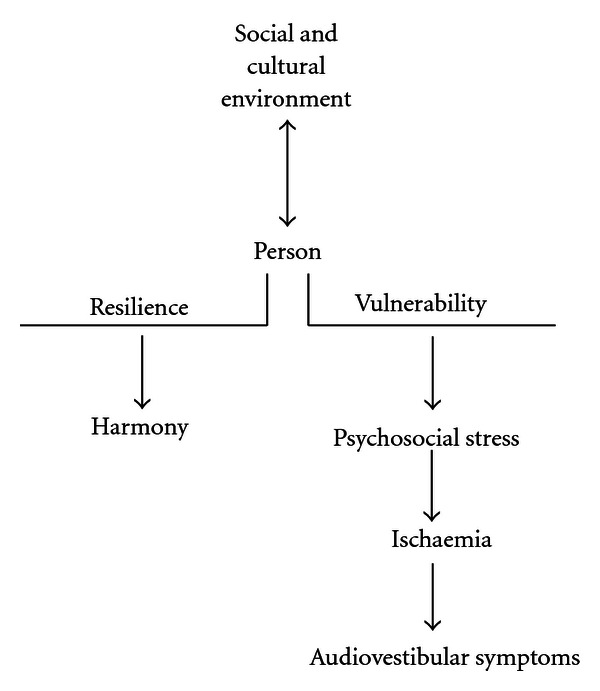
Social and cultural interactions of a person as generators of psychosocial stress. The interactions of the social and cultural environments with the person can produce diseases. The intensity of social and cultural stressors or their reiteration can undermine the resilience or resistance of a person, making him/her vulnerable. Psychosocial stress can generate a series of audiovestibular symptoms through the pathogenic mechanism of ischaemia (sudden deafness, Ménière disease, benign paroxysmal positional vertigo, tinnitus, and hyperacusis).

Figure 1 compiles the different aetiopathogenic mechanisms of ischaemia from the stress generated by interactions between the patient's social and cultural environment and the vulnerability or resiliency of a person. Blood analysis of the following substances: endothelin-1, prolactin, cortisol, adrenaline, noradrenaline, and cholesterol provides the means of an objective analysis of the patient's evolution.

### 2.3. Audiovestibular Symptoms Viewed from Otosociology

**Figure 2:**
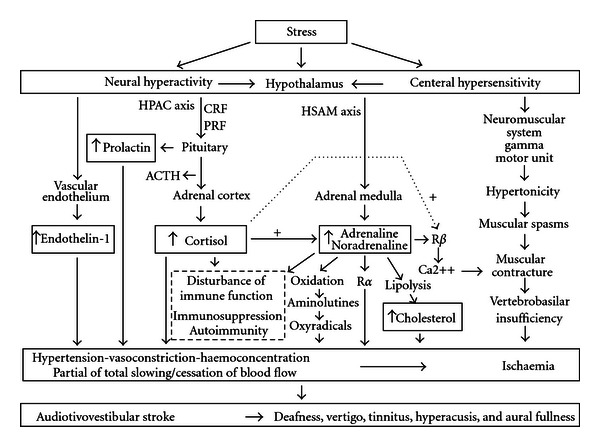
Mechanisms of action related to ischaemic stress that causes audiovestibular symptoms. ACTH: adrenocortical releasing factor. CRF: corticotrophin releasing factor. HPAC axis: hypothalamic-pituitary-adrenocortical axis. HSAM axis: hypothalamic-sympathetic-adrenomedullary axis. PRF: prolactin releasing factor. R*α*: receptor alpha. R*β*: receptor beta.

In light of the available literature on the subject that affirms that patients diagnosed with idiopathic sudden deafness have a higher probability of suffering a stroke in the next 5 years [42, 43], an otosociological viewpoint would indicate that otology lacks the necessary tools to consider these conditions from a more global perspective. With a mind-set focused on the ear, it is impossible to contemplate the triggering factors or causes that, of course, are not located within the ear, and so they continue to cause damage. Medicine attempts to improve a patient's health and environment in order to reach a better biological/psychological/social well-being, and so the expanded focus provided by otosociology comprehends the global reality of the patient much better, offering more chances of recovery. Otosociology understands that the causes of audiovestibular symptoms that patients describe are produced outside of the ear and even outside of the patient; that they originate in the patient's social environment. The social environment in which we carry out our lives implies situations that provoke states of anguish, anxiety, preoccupation, and irritability, as well as the sensation or perception of not being able to successfully confront these situations. This is what we commonly refer to as stress (Figure 2), which can be measured directly through blood levels of certain substances (endothelin-1, prolactin, cortisol, adrenaline, noradrenaline, and cholesterol), which allows for the objective analysis of its evolution.

As we have been discussing, the otologist treats sudden deafness, Ménière disease, and benign paroxysmal positional vertigo exclusively as otic diseases, whereas the otosociologist considers them to be symptoms of a cerebral ischaemic pathogenesis produced by psychosocial stress. That is to say, three different symptoms will all lead to the same diagnosis: audiovestibular stroke, with transient ischaemic auditory ictus commonly occurring in these cases.

In this manner, different studies have started to highlight the importance of lifestyle [44] and stress in the pathophysiological mechanisms of audiovestibular symptoms. Stress leads to vasoconstriction, haemoconcentration, and occlusion of the microcirculation that occurs in the inner ear [45]. Stress and stressful lifestyles, such as those related to work, social life, and emotional conflicts, as well as personality types that tend towards greater stress [46], have been correlated with audiovestibular symptoms. More specifically, studies relate situations of psychosocial stress with sudden deafness [47–49], Ménière disease [50], benign paroxysmal positional vertigo [51–54], tinnitus [55–64], and hyperacusis [65–67].

This theory is supported by the findings that social stress [1] can extend itself farther than simply one's social situation and can even produce physiological damage. In other fields of medicine, many diseases such as gastric ulcer [68, 69], diabetes [70], hypertension [71], and acute myocardial infarction [72] have a social component in their origins, whether from previous conditions [73] or sustained stress through time (chronic stress) [74].

After providing a detailed description of how otology defines, diagnoses, and treats that which it considers to be audiovestibular diseases, we have introduced the variable of ischaemia as an aetiopathogenic explanation, from the point of view of otosociology, of the true causes of these alterations. Alterations, not diseases, that have their origins outside of the ear and are external to the individual because they belong to the social environment. In this manner, we are now ready to expound upon and develop the specific field of otosociology in the following section.

## 3. Otosociology

We have already established that otosociology is a discipline dedicated to the “study, intervention, and prevention of organic and functional pathologies of the auditory system, with special emphasis on the influence of social factors.” As we can appreciate, otosociology stems from a holistic viewpoint that not only comprehends the dysfunction of an organ or body part, but rather the person in his/her entirety, including social and cultural environments. As such, it is evident that otosociology does not treat patients or subjects, but people. In this sense, otosociology involves a new theoretical framework with different names, concepts, and research processes from those used in the otological approach. The scientific basis of otosociology is based on scientific methodology of otolaryngology and sociology.

### 3.1. Origins

The history of human knowledge is full of situations in which two different people or disciplines come to similar conclusions, in the same period of time, given a certain problem. Serendipity, destiny, and chance have been used to describe this phenomenon which, in this case, brought together two different disciplines that researched a common subject; audiovestibular alterations, but with completely different objectives, methodologies, and points of view. They could even appear to be completely devoid of any connection to each other. In the case of medicine, the issue to be researched is based on finding an explanation for the causes of these alterations with the goal of elucidating an effective treatment; since viewed solely through the lens of biomedical methodology, the diagnosis is idiopathic and the treatment of symptoms alone is not sufficient to resolve this pathology. In the case of sociology, the common point consists of an unexpected finding in the study of the processes of exclusion of people with sudden deafness. In both cases, researchers were faced with a one-way alley: the causes of audiovestibular alterations were due to factors that were external to the biological states of individuals. They were due, in reality, to the stress produced by the individual's social and cultural environment. Collaboration for field work revealed the same conclusions that had been gained by observing the same individuals with different objectives. Thus commenced a scientific dialogue, with the end result of otosociology.

However, this description belies the complexity of the issue. The tight collaboration between two disciplines that differ significantly in their language, methods, and perspectives requires searching for points of common ground throughout the research process. Constant questions and clarifications arise regarding concepts, methodologies, and points of focus that are integral to one discipline but foreign to the other. It also requires substantial curiosity and respect for the pursuits of the other. This tension produced upon the collaboration of these two disciplines has the result of generating new ideas, but also highlights the fact that, in the majority of cases, the two groups are discussing the same issue but with different perspectives and languages. In any case, the experience can be very fruitful and beneficial to both parties. Integration of other ways of thinking and analysing problems based on common ground also requires significant effort and a learning curve from both sides, along with an expanded overall perspective and, above all, the possibility of doing the same things in different ways to reach results that, done in a different manner, would not have been achieved. Of course, during the process, frustration can arise as often as satisfaction, and many occurrences come to pass that are worth remembering.

Starting with the very first interviews performed with patients diagnosed with sudden deafness for sociological research, the collaboration commenced with a case by case discussion, providing different points of view and integrating new perspectives and viewpoints with each study interview, testing hypotheses. Case by case, and during a year and a half, weekly sessions lasting a mean of 5 hours gave way to the moulding and shaping of what was to become otosociology, which will be described in the following sections.

### 3.2. Professionals

Otosociologists must have a thorough understanding of the ear, but more importantly, must properly situate the ear within its surroundings. In this manner, an otosociologist is an otologist with training in sociology that takes into account the influence of social factors in dealing with patients. This involves performing not just an otological examination, but also a biosocial exploration of the patient and his/her condition. The otosociologist also collaborates with expert sociologists on otological issues.

Training in otosociology, which is given by otologists, sociologists, and psychologists, is directed towards doctors specialised in otorhinolaryngology through a focus on otosociology at the undergraduate or postgraduate level, using b-learning methodologies from sociology faculties, including study materials that delve into sociological theory, methodologies, and research techniques in addition to central themes related to audiovestibular processes.

### 3.3. Otosociological Methods and Materials

**Figure 3:**
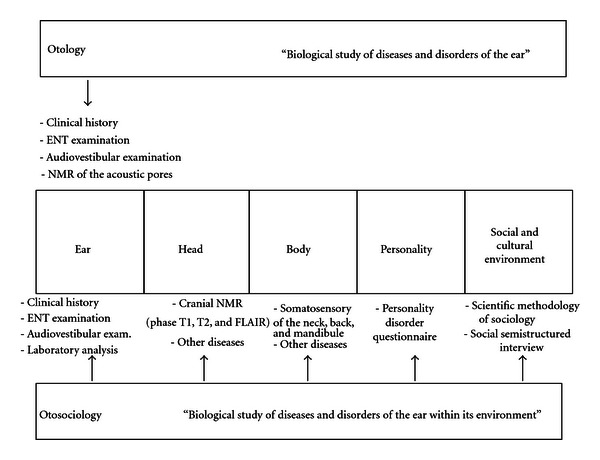
Methodology of otology and otosociology. Otology considers audiovestibular symptoms (deafness, vertigo, tinnitus, hyperacusis, and aural fullness) and places their aetiology, pathogenesis, diagnosis, and treatment within the ear. Otosociology places them within the ear and the individual's environment. ENT: ear-nose-throat. FLAIR: fluid attenuated inversion recovery. NMR: nuclear magnetic resonance.

As has been established, medicine involves its own scientific research methods and otologists utilise a series of protocols that they apply rigorously to their daily practice (Figure 3). Sociology boasts a qualitative method for investigation involving in-depth interviews, participant observation, and group discussions.

It is evident that the protocol currently used by biomedicine does not actually cure patients of what in otosociology we call audiovestibular alterations. It is also evident that the time allotted for a medical consultation is insufficient for applying the traditional qualitative methods employed in sociology. As such, otosociology utilises a hybrid method that combines the clinical and social condition of the patient, which the otosociologist can apply during the time allotted for a consultation.

 To this end, a pilot study lasting two months tested this hybrid methodology, which has allowed us to elaborate a specific, simplified protocol to be used in daily clinical practice by otosociologists. For the most complicated cases in which the protocol may be insufficient for detecting the causes that have led to the audiovestibular alterations, the otosociologist can seek the express collaboration of a sociologist that is also an expert in otology for an in-depth interview and later analysis.

In this pilot study, it was shown that an otologist with proper training in otosociology is capable, in an initial consultation lasting 20 minutes, of performing the normal clinical examination and applying otosociological protocols for arriving at the proper diagnosis and treatment. In cases that were harder to solve or in which the otosociologist had doubts regarding the causes of the alterations, a collaborating sociologist performed an in-depth interview and later compared conclusions with the otosociologist. In all cases, the response from the patient was positive with regard to the medical attention received, the explanations offered, the diagnosis made, and the treatment proposed.

The benefits of applying otosociology in otological consultations are many. The quality of the medical care given undergoes a substantial and objective improvement, and the perception of the patient is very positive. If we assess medical costs, these are considerably reduced by removing the need to prescribe unnecessary medication and repeated consultations. In terms of patient quality of life, we find that this improves immediately upon the consultation.

In the patients' self-evaluation of quality of life, one primary factor is that the person stops feeling like a patient, a sick person, and starts feeling like an individual capable of controlling the alterations that provoked the consultation. Additionally, the subject feels released from the years of dependency on medications and medical care, and finally, both he/she and his/her close friends and relatives understand what has occurred and its causes, facilitating over time a recovery from the alterations, whether partial or complete. Above all, intervening on the social and cultural causes of the issue removes the possibility that these same causes could repeat themselves in this or any other organ of the body. 

 The scientific basis will be imposed with the application of otosociological methodology.

## 4. Conclusions

Firstly, we can now count on collaborative teams between otology and sociology. We have reached a new mutual intellectual understanding through fluid communication and reciprocity. To this end, a positive disposition has been necessary for including other points of view and recognising complementary abilities, that is to say, being open to learning and adapting, maintaining the principle of respect for the capacities and contributions from both sides to concrete points as a whole.

Secondly we can also affirm that the collaboration between otologists and sociologists in health centres is possible in several different combinations. In an otorhinolaryngology department with one sociologist for every given number of otorhinolaryngologists, the otosociologist can resolve these cases by collaborating with the sociologist in those situations in which the social and cultural causes of the issue and proper interventions to treat it are not clear.

Thirdly, the integration of a sociologist and the training of otologists in otosociology will benefit all parties involved. The health system will benefit because its principles are centred on the needs of the patient, and the objectives therein are orientated towards promoting health and preventing disease, whether in conditions of treatment, recovery, or palliative care. We cannot forget either the economic efficiency in medication, diagnostic tests, and decreased repetition in doctor visits. The objective of the doctor is to cure, but this is often impossible in idiopathic diseases, which constitutes a source of frustration for medical practice. The otosociologist has the opportunity to elucidate the sociocultural causes that produces audiovestibular alterations and can carry out successful interventions to treat it, increasing doctor satisfaction and prestige. For the patient within the health system, otosociology provides high-quality medical care, demedicalisation of the issue, and a consequent recovery of the patient's identity as an individual, with substantial improvement in his/her condition. This all leads to improved perception of the multidimensional concept referred to as quality of life. By improving a person's concept of health, so too do their personal, family, and occupational relationships improve.

 To conclude, otosociology has arisen due to the inability of otology to provide effective solutions to the aforementioned audiovestibular symptoms. With this in mind, otosociology provides an aetiopathogenic explanation based on the cultural and social environment of the patient, delivering a correct diagnosis and definitive treatment.

## Figures and Tables

**Table 1:** General methodology of otology versus otosociology.

	Otology	Otosociology
Aetiology	Within the ear	In the individual's environment

Pathogenesis	Within the ear	Interaction between the individual and social environment

Diagnosis	Idiopathic	Based on evidence

Treatment	Symptomatic	Biopsychosocial

**Table 2:** Methodology in sudden deafness: otology versus otosociology.

	Otology	Otosociology
Aetiology	Within the ear	In the environment
	Inner ear	Social environment

Pathogenesis	Within the ear	Interaction
	Unknown	Stress, ischaemia

Diagnosis	Idiopathic	Based on evidence
	Idiopathic sudden deafness	Audiovestibular stroke

Treatment	Symptomatic	Biopsychosocial
	Medical: corticosteroids, vasodilators	Social, cultural, psychological, and medical

**Table 3:** Methodology in Ménière disease: otology versus otosociology.

	Otology	Otosociology
Aetiology	Within the ear	In the environment
	Inner ear	Social environment

Pathogenesis	Within the ear	Interaction
	Unknown	Stress, ischaemia

Diagnosis	Idiopathic	Based on evidence
	Ménière disease	Recurrent audiovestibular stroke

Treatment	Symptomatic	Biopsychosocial
	Medical: corticosteroids, vasodilators Surgical.	Social, cultural, psychological, and medical

**Table 4:** Methodology for benign paroxysmal positional vertigo: otology versus otosociology.

	Otology	Otosociology
Aetiology	Within the ear	In the environment
	Vestibule	Social environment

Pathogenesis	Within the ear	Interaction
	Displaced otoliths	Stress, ischaemia

Diagnosis	Idiopathic	Based on evidence
	Benign paroxysmal positional vertigo	Vestibular stroke

Treatment	Symptomatic	Biopsychosocial
	Otolith repositioning manoeuvres	Social, cultural, psychological, and medical
